# Supplementary material for: Transcriptional Effects of Psychoactive Drugs on Genes Involved in Neurogenesis
Source: Int J Mol Sci. 2020 Nov 6;21(21):8333. doi: 10.3390/ijms21218333 (PMC7672551; doi:10.3390/ijms21218333)
Supplement: Supplementary file 1 [file ijms-21-08333-s001.zip › Table A1.docx]

**Table A1.** The MANGO neurogenesis genes transcriptionally regulated by the 6 psychoactive drugs and corresponding processes.

| **Psychoactive**  **drug** | **Process** | **Genes regulated** |
| --- | --- | --- |
| Lithium | Axogenesis | *CCDC88A* |
|  | Dendritogenesis | *RASGRF1* |
|  | Differentiation | *CCND2, EGR1* |
|  | Expression | *VEGFA, SOX3, TUBA1A, EGR1, LGALS1, FABP3, CNTFR, RASGRF1, DGCR8, NPAS3* |
|  | Maturation | *EGR1* |
|  | Migration | *CCDC88A, PLCB1* |
|  | Numbers | *NPAS3, EPHA5, TAP1, ADRA2A, MYD88, CCND2, VEGFA, PLCB1, LGALS1, ST8SIA4, BCL2L11, BRINP1, EGR1, DGCR8* |
|  | Proliferation | *NPAS3, EPHA5, TAP1, ADRA2A, VEGFA, VGF, MYD88, CCND2, PLCB1, LGALS1, TUBA1A, BRINP1, EGR1, RASGRF1* |
|  | Survival | *NPAS3, VEGFA, RASGRF1* |
| Valproate | Dendritogenesis | *NOTCH1, MTOR, EPHB1, EPHB3, FGF2, FOXG1, WNT7A, LRRK2, CHD7, RASGRF1, ATP6AP2, CAMK2A, SNCA, PTN* |
|  | Differentiation | *SERPINE2, MECP2, NOTCH1, CTNNB1, PTPRF, BMP4, MBD1, EGF, NOG, FGF2, IGF2, FXR2, DRD2, RELA, WNT7A, SNCA, EGR1, EFNB2* |
|  | Expression | *NOTCH1, PER2, MMP2, CXCR4, SOX2, MSL1, SYN3, CDKN1A, EPHB1, EPHB3, ACHE, CTNNB1, CXCL12, CHRM4, TNFRSF1B, TNFRSF1A, SOX3, PTPRF, TUBA1A, FOS, EGR1, DPYSL3, CDK4, NDRG2, DBN1, FXR2, NOG, CALU, GSN, SNCA, GLI3, DRD2, LMNA, ACKR3, FABP3, SOX21, RELA, CNTFR, SOX4, CHD7, SMAD3, PTCH1, LEF1, RASGRF1, CORO1A, EFNB2, EPHB4, NPAS3, NGFR, NEFH, MECP2, CAMK2A, PTN* |
|  | Function | *CXCL12, GRIN2B, SMAD3, PTN* |
|  | Maturation | *EPHB1, EPHB3, GRIN2B, CREBBP, HTT, EGR1, SNCA* |
|  | Migration | *EPHB1, EPHB3, RELN, PLCB1, DCX, FOSB* |
|  | Neuritogenesis | *CTNNB1* |
|  | Numbers | *NOTCH1, NPAS3, PLAU, BMP4, SOX2, PER2, LIF, CDK5R1, NOG, CXCR4, CDKN1A, CDK2, EPHB1, ACHE, FGF2, NGF, TNFRSF1B, TNFRSF1A, FOXG1, PTPRF, RELN, GRM5, S100B, SNCA, MBD1, CTNNB1, GLP1R, PLCB1, CDK4, EGF, GSN, ST8SIA2, LRRK2, NOTCH2, FXR2, INHBA, BCL2L11, EPOR, SOX21, WNT7A, FOSB, SOX4, ODC1, DCX, LIPG, HTT, SMAD3, GRIN2B, EGR1, FXN, IGF2, CORO1A, EFNB2, E2F3, NGFR, MTOR, PTN* |
|  | Proliferation | *NOTCH1, NPAS3, SERPINE2, BMP4, SOX2, PLCG1, PER2, LIF, GRIN2B, MECP2, SYN3, CDKN1A, CDK2, EPHB1, VGF, ACHE, FGF2, NGF, TNFRSF1B, TNFRSF1A, FOXG1, PTPRF, RELN, S100B, SNCA, PGF, MCFD2, MBD1, GLP1R, PLCB1, CDK4, EGF, NOG, FXR2, DCX, LRRK2, INHBA, RAG1, DRD2, TOR1A, TUBA1A, GRIN2A, SOX21, CREBBP, WNT7A, ODC1, CHD7, DSP, HTT, EGR1, FXN, CTNNB1, RASGRF1, IGF2, CORO1A, NGFR, ATP6AP2, CAMK2A* |
|  | Survival | *NPAS3, SERPINE2, PER2, CXCR4, ACHE, NGF, PTPRF, SNCA, MCFD2, NOG, GSN, FXR2, INHBA, TOR1A, GRIN2A, CHD7, RASGRF1, CAMK2A* |
| Quetiapine | Axogenesis | *CCDC88A, NFKB1, NEUROD1* |
|  | Dendritogenesis | *NOTCH1, MTOR, GADD45B, BDNF, EPHB1, EPHB3, EFNB3, DISC1, NEUROD1, DKK1, NTRK2, WNT7A, CHD7, RASGRF1, CAMK2A, SNCA, SPARC* |
|  | Differentiation | *SERPINE2, FAS, ASCL1, MECP2, NOTCH1, CTNNB1, PTPRF, BMP4, CCND2, NEUROD1, TLR3, GDNF, STAT3, GPX1, NOG, DRD2, BDNF, RELA, WNT7A, SNCA, DNMT1, EGR1, EFNB2* |
|  | Expression | *NOTCH1, VEGFA, NEUROD1, ARC, ASCL1, MMP2, HOPX, CXCR4, SOX2, LEPR, SYN3, NTRK2, CDKN1A, JAG1, EPHB1, EPHB3, EPHB2, ACHE, CASP7, CTNNB1, CXCL12, SOX3, ABCA2, PTPRF, CNR1, CCND3, CCND1, NES, CALB2, EGR1, NOS1, DPYSL3, CST3, CDK6, EPHA4, DBN1, NOG, CALU, RRM1, ATF2, SNCA, GLI2, GLI3, GRIA2, DRD2, LMNB1, FABP3, RDX, BMPR1A, BIRC5, RELA, HBEGF, AR, CNTFR, SOX4, NAMPT, DNMT1, CHD7, CACNA1C, PTCH1, IFNAR1, RASGRF1, HMGB2, DRAXIN, DCC, CORO1A, EFNB2, NPAS3, NGFR, SYP, MECP2, LAMB1, SLC1A2, CAMK2A, SPARC, PTPRZ1* |
|  | Function | *CXCL12, GRIN2B, NTRK2, DISC1, NEUROD1* |
|  | Maturation | *EPHB1, EPHB3, DISC1, GRIN2B, CREBBP, BDNF, EGR1, SNCA* |
|  | Migration | *CCDC88A, BDNF, EPHB1, EPHB3, EFNB3, DISC1, RELN* |
|  | Neuritogenesis | *CTNNB1* |
|  | Numbers | *TLR3, THRA, NOTCH1, NPAS3, BMPR1A, CNR1, EPHA5, TAP1, NPY, BMP4, SOX2, SLC30A3, NEUROD1, STAT3, NOG, BDNF, HOPX, CXCR4, DLST, DLD, NFKB1, SORL1, CDKN1A, EPHB1, ACHE, MYD88, NOS1, FGFR1, CIT, TGFB1, NTF3, RARA, PTPRF, RELN, BCL2, GDNF, FAAH, SNCA, CCND2, VEGFA, NTRK2, E2F1, CST3, CTNNB1, CDK6, CCND1, GPX1, DNMT1, ST8SIA2, TET1, NOTCH2, INHBA, FST, BBC3, TGFBR2, MAP2K5, BIRC5, ARNTL, WNT7A, HBEGF, BRAF, BRINP1, LEPR, RB1CC1, SOX4, ODC1, NAMPT, BTG1, GRIN2B, PRKDC, EGR1, FXN, IFNAR1, NR1D1, DRAXIN, CORO1A, ASCL1, EFNB2, NGFR, MTOR* |
|  | Proliferation | *THRA, NOTCH1, BMPR1A, NPAS3, TGFB1, CNR1, SERPINE2, EPHA5, BCL2L1, TAP1, NFKB1, NPY, BMP4, SOX2, FAS, SLC30A3, PLCG1, GADD45B, FGF1, STAT3, BDNF, VEGFA, GRIN2B, MECP2, HOPX, SORL1, SYN3, CDKN1A, EPHB1, EFNB3, VGF, SLC7A11, ACHE, MYD88, NOS1, FGFR1, NTF3, RARA, PTPRF, RELN, BCL2, AR, SNCA, CCND2, SPP1, HBEGF, MCFD2, E2F1, NTRK2, CDK6, CCND1, GPX1, NOG, DKK1, EPHB2, RHOA, TET1, GDNF, LRP2, INHBA, FST, DRD2, TOR1A, BIRC5, CREBBP, ARNTL, WNT7A, BRAF, BRINP1, LEPR, ODC1, DNMT1, CHD7, DSP, DICER1, EGR1, BTG1, FXN, CTNNB1, RASGRF1, CORO1A, ASCL1, NGFR, CAMK2A* |
|  | Survival | *THRA, TLR3, NPAS3, CNR1, SERPINE2, BDNF, HOPX, CXCR4, NFKB1, SORL1, ACHE, RARA, PTPRF, BCL2, NPY, SNCA, VEGFA, NTRK2, MCFD2, CASP9, GPX1, NOG, DKK1, RHOA, NOS1, INHBA, FST, TOR1A, DNMT1, CHD7, BTG1, RASGRF1, CAMK2A* |
| Amisulpride | Dendritogenesis | *APOE* |
|  | Differentiation | *APOE, GPX1* |
|  | Expression | *GRIN1, CST3, NOTCH3, APOE* |
|  | Maturation | *APOE* |
|  | Numbers | *CST3, GPX1, GRIN1, MAP2K5, NOTCH3* |
|  | Proliferation | *VGF, GRIN1, GPX1, NOTCH3* |
|  | Survival | *GPX1* |
| Aripiprazole | Axonogenesis | *CCDC88A* |
|  | Dendritogenesis | *NOTCH1, APOE* |
|  | Differentiation | *APOE, NOTCH1* |
|  | Expression | *NOTCH1, MMP2, CST3, MYCN, LMNB2, NOTCH3, APOE, EPHB4* |
|  | Maturation | *APOE* |
|  | Migration | *CCDC88A* |
|  | Numbers | *NOTCH1, NGF, CST3, BBC3, NOTCH3* |
|  | Proliferation | *NOTCH1, NGF, NOTCH3* |
|  | Survival | *NGF* |
| Clozapine | Dendritogenesis | *NOTCH1, GADD45B, APOE, SPARC, DBI* |
|  | Differentiation | *APOE, NOTCH1, CCND2, EGF, GDNF, GPX1, IGF2* |
|  | Expression | *NOTCH1, MMP2, SOX2, LEPR, JAG1, SOX3, CCND1, NES, CALB2, CST3, CDK6, GLI2, LMNB2, FABP3, SOX21, HBEGF, NOTCH3, APOE, EPHB4, LAMB1, SPARC, DBI* |
|  | Maturation | *APOE* |
|  | Numbers | *NOTCH1, SOX2, FGFR1, GDNF, CCND2, CST3, CDK6, CCND1, EGF, GPX1, NOTCH2, INHBA, SOX21, HBEGF, LEPR, NOTCH3, IGF2* |
|  | Proliferation | *NOTCH1, SOX2, GADD45B, FGFR1, CCND2, HBEGF, CDK6, CCND1, EGF, GPX1, GDNF, INHBA, SOX21, LEPR, NOTCH3, IGF2* |
|  | Survival | *GPX1, INHBA* |
