# Supplementary material for: Transcriptional Effects of Psychoactive Drugs on Genes Involved in Neurogenesis
Source: Int J Mol Sci. 2020 Nov 6;21(21):8333. doi: 10.3390/ijms21218333 (PMC7672551; doi:10.3390/ijms21218333)
Supplement: Supplementary file 1 [file ijms-21-08333-s001.zip › Table A2.docx]

**Table A2:** Transcription factors predicted to affect the expression of genes in the MANGO database.

|  | **TF** | **OLG** | **P value** | **Q value** |
| --- | --- | --- | --- | --- |
| RELA | RELA | 24 | 5.67E-18 | 4.93E-16 |
| NFKB1 | NFKB1 | 24 | 6.61E-18 | 4.93E-16 |
| SP1 | SP1 | 27 | 1.81E-16 | 9.01E-15 |
| TP53 | TP53 | 18 | 3.79E-16 | 1.41E-14 |
| STAT3 | STAT3 | 17 | 5.96E-16 | 1.78E-14 |
| HDAC1 | HDAC1 | 13 | 6.14E-15 | 1.53E-13 |
| E2F1 | E2F1 | 14 | 1.67E-12 | 3.56E-11 |
| EGR1 | EGR1 | 10 | 1.23E-09 | 2.29E-08 |
| MYC | MYC | 10 | 4.37E-09 | 7.24E-08 |
| ESR1 | ESR1 | 9 | 5.89E-09 | 8.77E-08 |
| EP300 | EP300 | 8 | 9.29E-09 | 1.26E-07 |
| NR3C1 | NR3C1 | 7 | 1.28E-08 | 1.59E-07 |
| FHL2 | FHL2 | 4 | 1.57E-08 | 1.80E-07 |
| KLF4 | KLF4 | 7 | 1.87E-08 | 1.99E-07 |
| PARP1 | PARP1 | 6 | 2.68E-08 | 2.66E-07 |
| RB1 | RB1 | 6 | 1.07E-07 | 9.39E-07 |
| SMAD3 | SMAD3 | 6 | 1.07E-07 | 9.39E-07 |
| BCL6 | BCL6 | 5 | 1.34E-07 | 1.11E-06 |
| FOXO3 | FOXO3 | 5 | 1.84E-07 | 1.44E-06 |
| JUN | JUN | 10 | 2.01E-07 | 1.47E-06 |
| ATF5 | ATF5 | 4 | 2.16E-07 | 1.47E-06 |
| HIF1A | HIF1A | 8 | 2.17E-07 | 1.47E-06 |
| FOS | FOS | 7 | 2.38E-07 | 1.54E-06 |
| VHL | VHL | 5 | 3.29E-07 | 2.04E-06 |
| CTNNB1 | CTNNB1 | 5 | 5.52E-07 | 3.29E-06 |
| POU5F1 | POU5F1 | 5 | 8.81E-07 | 5.05E-06 |
| SP4 | SP4 | 4 | 1.00E-06 | 5.52E-06 |
| PGR | PGR | 5 | 1.09E-06 | 5.62E-06 |
| TCF4 | TCF4 | 5 | 1.09E-06 | 5.62E-06 |
| KLF5 | KLF5 | 4 | 2.14E-06 | 1.03E-05 |
| TP73 | TP73 | 4 | 2.14E-06 | 1.03E-05 |
| SP3 | SP3 | 8 | 2.33E-06 | 1.08E-05 |
| YBX1 | YBX1 | 5 | 2.85E-06 | 1.29E-05 |
| ATF2 | ATF2 | 5 | 3.97E-06 | 1.72E-05 |
| ING4 | ING4 | 4 | 4.04E-06 | 1.72E-05 |
| ZNF24 | ZNF24 | 3 | 4.21E-06 | 1.74E-05 |
| BRCA1 | BRCA1 | 6 | 4.49E-06 | 1.76E-05 |
| WT1 | WT1 | 6 | 4.49E-06 | 1.76E-05 |
| CREB1 | CREB1 | 7 | 5.46E-06 | 2.09E-05 |
| AR | AR | 7 | 6.79E-06 | 2.53E-05 |
| SOX6 | SOX6 | 3 | 8.38E-06 | 3.05E-05 |
| PPARG | PPARG | 6 | 1.06E-05 | 3.77E-05 |
| TFAP2A | TFAP2A | 6 | 1.63E-05 | 5.63E-05 |
| ATM | ATM | 4 | 2.08E-05 | 6.88E-05 |
| ETV4 | ETV4 | 4 | 2.08E-05 | 6.88E-05 |
| MYCN | MYCN | 5 | 2.23E-05 | 7.22E-05 |
| ERCC2 | ERCC2 | 3 | 2.32E-05 | 7.36E-05 |
| NANOG | NANOG | 4 | 2.50E-05 | 7.61E-05 |
| SOX9 | SOX9 | 4 | 2.50E-05 | 7.61E-05 |
| HIC1 | HIC1 | 3 | 3.46E-05 | 0.000103 |
| IRF1 | IRF1 | 5 | 4.13E-05 | 0.000119 |
| NR1I2 | NR1I2 | 4 | 4.15E-05 | 0.000119 |
| HDAC2 | HDAC2 | 4 | 4.84E-05 | 0.000126 |
| ABL1 | ABL1 | 3 | 4.92E-05 | 0.000126 |
| HOXB7 | HOXB7 | 3 | 4.92E-05 | 0.000126 |
| NKX3-1 | NKX3-1 | 3 | 4.92E-05 | 0.000126 |
| TFAP2C | TFAP2C | 3 | 4.92E-05 | 0.000126 |
| ZNF382 | ZNF382 | 3 | 4.92E-05 | 0.000126 |
| LEF1 | LEF1 | 4 | 6.48E-05 | 0.000164 |
| NPM1 | NPM1 | 3 | 6.72E-05 | 0.000167 |
| RUNX3 | RUNX3 | 4 | 7.43E-05 | 0.000181 |
| DNMT1 | DNMT1 | 4 | 8.48E-05 | 0.000201 |
| HSF1 | HSF1 | 4 | 8.48E-05 | 0.000201 |
| APC | APC | 3 | 8.92E-05 | 0.000204 |
| TCF7L2 | TCF7L2 | 3 | 8.92E-05 | 0.000204 |
| NFYC | NFYC | 3 | 0.000115 | 0.000256 |
| STAT5A | STAT5A | 3 | 0.000115 | 0.000256 |
| JUND | JUND | 4 | 0.000123 | 0.000269 |
| NFYB | NFYB | 3 | 0.000146 | 0.000306 |
| SNAI2 | SNAI2 | 3 | 0.000146 | 0.000306 |
| TCF3 | TCF3 | 3 | 0.000146 | 0.000306 |
| TBP | TBP | 3 | 0.000181 | 0.000375 |
| RUNX1 | RUNX1 | 4 | 0.000234 | 0.000477 |
| GLI2 | GLI2 | 3 | 0.00032 | 0.000644 |
| ETS1 | ETS1 | 5 | 0.000334 | 0.000664 |
| NFKBIA | NFKBIA | 3 | 0.000378 | 0.000731 |
| RUNX2 | RUNX2 | 3 | 0.000378 | 0.000731 |
| NFYA | NFYA | 3 | 0.000442 | 0.000828 |
| NR1H4 | NR1H4 | 3 | 0.000442 | 0.000828 |
| STAT1 | STAT1 | 5 | 0.000444 | 0.000828 |
| SIRT1 | SIRT1 | 4 | 0.000475 | 0.000873 |
| PAX5 | PAX5 | 3 | 0.000513 | 0.00092 |
| BCL3 | BCL3 | 2 | 0.000562 | 0.00092 |
| ETV6 | ETV6 | 2 | 0.000562 | 0.00092 |
| KDM4B | KDM4B | 2 | 0.000562 | 0.00092 |
| NUPR1 | NUPR1 | 2 | 0.000562 | 0.00092 |
| RORA | RORA | 2 | 0.000562 | 0.00092 |
| SMAD2 | SMAD2 | 2 | 0.000562 | 0.00092 |
| TAF1 | TAF1 | 2 | 0.000562 | 0.00092 |
| TNFAIP3 | TNFAIP3 | 2 | 0.000562 | 0.00092 |
| TSC22D3 | TSC22D3 | 2 | 0.000562 | 0.00092 |
| SOX2 | SOX2 | 3 | 0.00059 | 0.000956 |
| E2F4 | E2F4 | 3 | 0.000675 | 0.00108 |
| FOXO1 | FOXO1 | 3 | 0.000767 | 0.0012 |
| HDAC3 | HDAC3 | 3 | 0.000767 | 0.0012 |
| HDGF | HDGF | 2 | 0.000839 | 0.00128 |
| NFKB2 | NFKB2 | 2 | 0.000839 | 0.00128 |
| PAX3 | PAX3 | 2 | 0.000839 | 0.00128 |
| SMAD4 | SMAD4 | 3 | 0.000975 | 0.00147 |
| ERG | ERG | 3 | 0.00109 | 0.00161 |
| SREBF1 | SREBF1 | 3 | 0.00109 | 0.00161 |
| APEX1 | APEX1 | 2 | 0.00117 | 0.00167 |
| HOXA1 | HOXA1 | 2 | 0.00117 | 0.00167 |
| KLF10 | KLF10 | 2 | 0.00117 | 0.00167 |
| USF1 | USF1 | 4 | 0.0015 | 0.0021 |
| HES1 | HES1 | 2 | 0.00155 | 0.0021 |
| IRF2 | IRF2 | 2 | 0.00155 | 0.0021 |
| IRF9 | IRF9 | 2 | 0.00155 | 0.0021 |
| MEF2C | MEF2C | 2 | 0.00155 | 0.0021 |
| STAT5B | STAT5B | 2 | 0.00155 | 0.0021 |
| ETS2 | ETS2 | 3 | 0.0018 | 0.00239 |
| REST | REST | 3 | 0.0018 | 0.00239 |
| BTF3 | BTF3 | 2 | 0.00198 | 0.00253 |
| HIPK2 | HIPK2 | 2 | 0.00198 | 0.00253 |
| HOXA9 | HOXA9 | 2 | 0.00198 | 0.00253 |
| SMAD7 | SMAD7 | 2 | 0.00198 | 0.00253 |
| SMARCA4 | SMARCA4 | 2 | 0.00198 | 0.00253 |
| TWIST1 | TWIST1 | 3 | 0.00234 | 0.00295 |
| EPAS1 | EPAS1 | 2 | 0.00247 | 0.00306 |
| ZBTB16 | ZBTB16 | 2 | 0.00247 | 0.00306 |
| POU2F1 | POU2F1 | 3 | 0.00296 | 0.0036 |
| KLF2 | KLF2 | 2 | 0.003 | 0.0036 |
| MECP2 | MECP2 | 2 | 0.003 | 0.0036 |
| MSC | MSC | 2 | 0.003 | 0.0036 |
| EZH2 | EZH2 | 3 | 0.00343 | 0.00409 |
| E2F3 | E2F3 | 2 | 0.00421 | 0.00498 |
| NR4A1 | NR4A1 | 2 | 0.00561 | 0.00648 |
| PTTG1 | PTTG1 | 2 | 0.00561 | 0.00648 |
| SATB1 | SATB1 | 2 | 0.00561 | 0.00648 |
| CEBPA | CEBPA | 3 | 0.0068 | 0.0078 |
| FOXM1 | FOXM1 | 2 | 0.0072 | 0.00819 |
| HDAC9 | HDAC9 | 2 | 0.00806 | 0.0091 |
| ESR2 | ESR2 | 2 | 0.00896 | 0.00989 |
| GLI1 | GLI1 | 2 | 0.00896 | 0.00989 |
| PML | PML | 2 | 0.00896 | 0.00989 |
| KLF6 | KLF6 | 2 | 0.00991 | 0.0109 |
| REL | REL | 2 | 0.0119 | 0.013 |
| HDAC4 | HDAC4 | 2 | 0.0141 | 0.0151 |
| MTA1 | MTA1 | 2 | 0.0141 | 0.0151 |
| CREBBP | CREBBP | 2 | 0.0153 | 0.016 |
| SRF | SRF | 2 | 0.0153 | 0.016 |
| TWIST2 | TWIST2 | 2 | 0.0153 | 0.016 |
| CTCF | CTCF | 2 | 0.0216 | 0.0225 |
| ATF4 | ATF4 | 2 | 0.0288 | 0.0298 |
| STAT6 | STAT6 | 2 | 0.0304 | 0.0312 |
| YY1 | YY1 | 3 | 0.0319 | 0.0326 |
| GATA3 | GATA3 | 2 | 0.0336 | 0.034 |
| USF2 | USF2 | 2 | 0.0494 | 0.0497 |

*TF=transcription factor, OLG=overlapping genes.*
