# Supplementary figures and images for: Transcriptional Effects of Psychoactive Drugs on Genes Involved in Neurogenesis
Source: Int J Mol Sci. 2020 Nov 6;21(21):8333. doi: 10.3390/ijms21218333 (PMC7672551; doi:10.3390/ijms21218333)

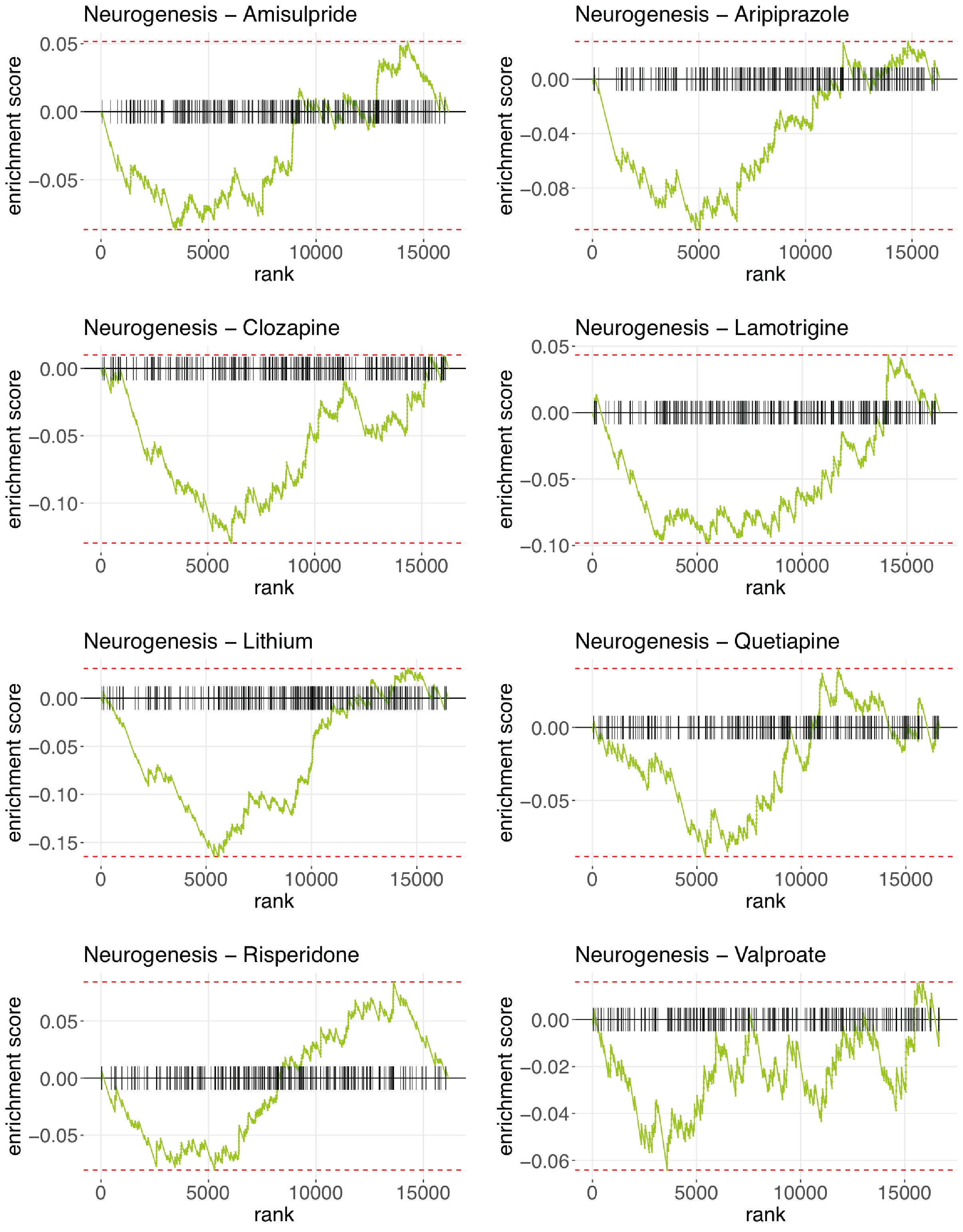

Supplement: Supplementary file 1 [file ijms-21-08333-s001.zip › fig a1.png]
